# Interventions for children's language and literacy difficulties

**DOI:** 10.1111/j.1460-6984.2011.00081.x

**Published:** 2012-01

**Authors:** Margaret J Snowling, Charles Hulme

**Affiliations:** Department of Psychology, University of YorkYork, UK

**Keywords:** decoding, reading comprehension, dyslexia, poor comprehender, reading intervention, language intervention

## Abstract

Against a backdrop of research on individual differences in reading disorders, this review considers a range of effective interventions to promote reading and language skills evaluated by our group. The review begins by contrasting the reading profiles seen in dyslexia and reading comprehension impairment and then argues that different interventions will be required. It is well established that effective interventions for decoding deficits (dyslexia) involve work on letter–sound knowledge, phonological awareness and reading practice to reinforce emergent skills. In contrast, effective interventions for reading comprehension difficulties involve training to promote oral language skills and text comprehension strategies. Together the findings of controlled trials provide a robust evidence base that can be used to devise plans for the management of pre-school and school-aged children with language learning difficulties.

**What this paper adds**

This review shows that problems with word-level decoding are distinct from problems of reading comprehension. Different interventions are required to promote decoding and reading comprehension skills. Evidence-based approaches to promote decoding involve training in letter knowledge, phoneme awareness and linking these during text reading. To promote reading comprehension, approaches which work directly on text comprehension strategies and on oral language skills are effective, with vocabulary instruction being a particularly important technique.

## Introduction

In their classic text *Language by Ear and by Eye*, Kavanagh and Mattingley (1972) explored the relationships between ‘speech and learning to read’. It was claimed that ‘reading is parasitic upon speech’, and this simple idea has dominated the field for many years. However, it is increasingly clear that it is a narrow view. Aside from children who ‘can listen and speak well’, yet have difficulty learning to read (dyslexia), the main risk factor for reading disorders is problematic language development. It follows that speech and language therapists play a critical role in the identification of children who are likely to go on to have literacy difficulties and should be well positioned to provide management advice. The present review begins by outlining the relationships between language and reading difficulties before proceeding to discuss recent evidence-based interventions to promote language and literacy skills evaluated by our group.

## The nature of reading disorders

Reading is a complex skill that can dissociate to produce varying profiles of impairment (Bishop and Snowling 2004). The two most common forms of reading disorder are dyslexia, a specific difficulty with decoding print, and reading comprehension impairment, a specific difficulty with text comprehension. Dyslexia was first described at the end of the 19th century and has been the subject of scientific research for more than 40 years (Snowling 2009, Vellutino *et al.* 2004). In contrast, reading comprehension impairment (often referred to as the poor comprehender profile) has attracted much less research since it was first described during the 1980s (Oakhill 1984), although a robust empirical base is now emerging (Cain 2010). Poor comprehenders are often characterized as having a hidden handicap because they decode well and, on the surface, are fluent readers. It is only when they are asked questions about what they have read that their difficulties are revealed. Poor comprehenders are also neglected in the *Diagnostic and Statistical Manual* (DSM-IV) of the American Psychiatric Association (1994) which does not recognize reading comprehension impairment as a separate category of reading disorder. Moreover, in the draft version of the fifth edition of the *Diagnostic and Statistical Manual* (http://www.dsm5.org), specific reading comprehension impairment is not listed as a form of reading disorder; rather it is considered to be a form of language impairment. It is clearly important for children with poor reading comprehension to be given better recognition within the school system where, at present, their support needs are largely unmet.

Disorders of reading are relatively common in mainstream schools and this provides a strong case for interventions not only for decoding difficulties but also for reading comprehension impairments. The approach to the two disorders needs to be distinct and to take account of the underlying nature of these difficulties. It also needs to draw on evidence of the efficacy of treatments. This paper begins by reviewing the principles that are now well established for interventions that target basic decoding skills before going on to discuss effective interventions to promote reading comprehension. It also considers early interventions at the foundations of reading skill and suggests these can be an important step toward reducing the number of poor readers in the school-aged population.

## Interventions for language and reading

Hulme and Snowling (2009) have emphasized that a good starting point for developing an intervention is a causal theory. Within this view, the causes of a reading disorder provide the theoretical motivation for the design and content of an intervention; furthermore, the findings from an intervention study will provide a test of the causal theory. This simple but important point underlines what was stated some 30 years ago by Bradley and Bryant (1983)—that the positive effects of training phonological awareness on reading skill provides a ‘proof’ of the causal role of phonological awareness in reading development. As shall be shown below, this causal theory has in fact been modified in the light of evidence that interventions that train phonological awareness alone are less effective than those which link emergent phonological awareness with letter–sound knowledge in the service of reading (Hatcher *et al.* 1994). Thus, the findings of applied research can be used to test a theory and to feedback and so modify it (a so-called virtuous circle). The interventions that will be considered have been designed to target skills known to be deficient in poor readers and their findings have been used not only to inform educational practice, but also to test and refine casual theories.

### Interventions to promote word level decoding and fluency

The main ingredients of a teaching approach to promote word-level decoding skills is one that combines training in phonological awareness with training in letter–sound knowledge and in which these two skills are reinforced in the context of reading (Torgesen 2005, Snowling and Hulme 2011). Such an approach goes beyond the contemporary emphasis on systematic ‘phonics’ by ensuring that children have adequate phonological awareness skills and by ensuring that what is taught is practised in context which, in turn, can provide a vital bootstrapping resource for children who have significant phonological difficulties.

Hatcher *et al.* (1994) were the first group of researchers in the UK to assess the efficacy of different forms of intervention for readers with dyslexic profiles using a controlled design. Children participating in this study were identified through a countywide screening of all children in their third year in school. Those selected to take part in the study had reading skills that fell within the bottom 10% of the population in terms of reading accuracy. The children were then randomly allocated to one of four experimental conditions. In three conditions the children received intervention, the fourth was a control group that received ‘business as usual’.

The three interventions that were trialled were theoretically motivated and based on best practice at the time (Bradley and Bryant 1983, Clay 1985, Lundberg 1994). The interventions were delivered on a twice weekly basis by skilled teachers for 20 weeks. Each teacher taught in each arm of the intervention to control for the quality of delivery. There were three interventions: reading alone (R) in which children read from texts which were selected to be at the appropriate level and teachers reinforced effective reading strategies to hone the children's skills (for further details, see Hatcher 2006). The second condition was phonology alone (P), which consisted of exercises training the development of oral phonological awareness at syllable, rhyme and phoneme levels following the ideas of Bradley and Bryant (1983) but not involving letter work. The third intervention, reading with phonology (R+P), combined the reading and phonology approaches. The children receiving this intervention where trained in phonological awareness and letter–sound knowledge and were encouraged through the reading of texts at the easy and instructional levels to practise their emergent skills.

At the end of the 20 weeks of intervention the children who received the combined programme (R+P) were significantly ahead of the other three groups in reading accuracy, spelling and reading comprehension. These gains in reading were maintained 5 months after the intervention ceased, but at this stage the benefits of spelling had weakened (it should be noted that spelling was not explicitly taught within the programme). At the time these findings challenged the theory that phonological deficits alone cause reading impairment, since it was only when phonological awareness was trained in the context of orthography that the impact on reading was significant. Indeed, children who received the phonology alone (P) programme were ahead of the others in phonological awareness at the end of the intervention but these gains had not generalized to their literacy skills.

The work of Hatcher *et al.* (1994) has been influential in terms not only of theory, but also of practice, and they have influenced the direction of subsequent intervention trials conducted by our group. First, the principles and the sequence of activities used in the R+P programme informed a classroom-based study using mainstream teachers (Hatcher *et al.* 2004) and subsequently a modified version of the R+P programme was designed for implementation by trained teaching assistants (Hatcher *et al.* 2006a). This new programme was next evaluated in a randomized controlled trial which for children in Year 1 with reading difficulties (Hatcher *et al.* 2006b). The programme was delivered on a daily basis by teaching assistants, alternating between group and individual sessions. During the group session children worked in groups of three on activities to promote phonological awareness, letter–sound knowledge and sound linkage activities including writing a simple story. In the individual sessions the work focused on reading using both an easy book and a book with the instructional level determined through the teacher's use of a running record (Clay 1985). The children were randomly allocated to receive the intervention for either 20 or 10 weeks. The experimental group received the intervention for 10 weeks in the spring term and for 10 weeks in the summer term, while the waiting control group only received the intervention during 10 weeks in the summer term. This meant that for the first 10 weeks the impact of the intervention could be gauged against ‘business as usual’.

The findings of the intervention were extremely encouraging. There was a clearly significant effect of intervention on reading accuracy scores on a standardized test; the gains made were over 7 standard score points during the 20 weeks of the intervention. This rate of improvement can be regarded as educationally significant and is comparable with that found in other studies internationally.

### Early intervention to circumvent decoding difficulties

An obvious question that follows from the successful implementation of interventions to promote decoding skills is why wait for failure? A great deal is known about what places a child at risk of reading difficulties and hence there would seem to be no good reason to wait until a child has failed before implementing a remediation programme.

With this in mind, Bowyer-Crane *et al.* (2008) evaluated a 20-week intervention programme using the principles of the R+P intervention for children who entered school with poor speech and language development. The programme was a modification of that used by Hatcher *et al.* (2006b) designed to be suitable for younger children, and hence renamed the P+R programme. It comprised three main components: letter–sound work, segmenting and blending, and reading together and reading independently. Once again it alternated between group and individual sessions on a daily basis. Four children worked together in a group on letter–sound knowledge, segmenting and blending and in the individual sessions the work focused on reading but also incorporated work to reinforce knowledge of letters and sounds. A more detailed breakdown of the activities is shown in Table 1.

**Table 1:** Outline of phonology and reading (P+R) sessions (Bowyer-Crane *et al.* 2008)

Group session (30 min)	Individual session (20 min)
Introduction (4 min)	Introduction (2 min)
New letter introduction (8 min)	Working with sounds (3 min)
Group book work (8 min)	Sight word learning (5 min)
Segmentation or blending (5 min)	Reading Books (10 min):
Plenary (5 min)	Running record
	Introduce new book

The children who took part in the intervention were identified following screening in 23 large schools on the basis of their poor performance on tests of expressive and receptive vocabulary. The mean age of the children was 4 years 10 months (standard deviation = 3.3 months) and their language levels were at roughly the 10th centile for their age. Twenty-four per cent of the sample was in receipt of free school meals and some 22.6% were rated by their teachers as having behavioural difficulties.

The intervention was implemented by trained teaching assistants who were supported throughout the intervention in fortnightly tutorials (for further details, see Carroll *et al.* 2011). These same teaching assistants also delivered an oral language intervention (OL) in the same classrooms. The children in the two intervention programmes were assessed before the intervention, mid-intervention after 10 weeks and post-intervention after 20 weeks. To assess maintenance of gains they were also seen 5 months after the intervention had finished.

To evaluate the efficacy of the P+R programme, the gains of the children on tests of reading and reading related skills were compared with those of the treated control group who received the OL intervention. These analyses took account of the clustering of children within schools as well as baseline performance on each of the dependent measures. Since there was no untreated control group in this study, the findings are conservative.

The results of the intervention were very encouraging. The outcomes for the children who had received the P+R intervention were significantly better than those in the OL group for prose reading accuracy, non-word reading, spelling, and segmenting and blending. In addition, gains for single-word reading were marginally significant. It is possible to express these gains in terms of effect size: letter knowledge, *d*= 0.4; spelling, *d*= 0.4; non-word reading, *d*= 0.4; prose reading accuracy, *d*= 0.4; and segmenting and blending, *d*= 0.7. Moreover, comparison of the outcomes of these children in relation to a large sample of 700 classroom peers 5 months after the intervention were pleasing with more than 50% now performing within the average range for early word-reading skills (7% had standard reading scores above 115).

In summary, training children in phoneme segmentation and blending and reinforcing this work through reading activities was an effective intervention for promoting basic reading skills for children who entered school with poorly developed speech and language. The effect of the intervention were maintained 5 months after it ceased and it is perhaps important to note that the gains generalized to non-word reading, a pure test of decoding. Preliminary analysis suggests that the benefits of the P+R programme on reading were mediated by gains in phoneme awareness and letter knowledge (Hulme, in preparation). Thus, it is possible to improve significantly the reading skills of children who show poor decoding (dyslexia) by systematic training in phoneme awareness, letter–sound knowledge and reading.

### Interventions to promote reading comprehension

In many ways, the cognitive profile associated with reading comprehension impairment directly contrasts with that associated with dyslexia. Whereas children with dyslexia have pervasive phonological deficits, phonological skills are typically normal in poor comprehenders (Nation 2005). In contrast, reading comprehension impairment is associated with a wide range of language processing difficulties, for example with grammar and sentence structure, and for many of these children vocabulary knowledge is poor. Children with reading comprehension impairment also experience a range of difficulties with aspects of text processing. In particular, they have difficulty making inferences that link sentences and make texts cohere (Cain *et al.* 2000), and they have difficulty in monitoring the sense of what they are reading and in using metacognitive strategies such as looking back on the text to resolve ambiguity. Against this backdrop, it is not surprising that many intervention studies have targeted higher level text comprehension skills such as making inferences (Yuill and Oakhill 1988, Yuill and Joscelyne 1988) as well incorporating the use of visual imagery to enhance the representation of text (Oakhill and Patel 1991, Joffe *et al.* 2007). However, until recently the majority of intervention studies for poor comprehenders have been relatively small in scale and have not used random allocation of children to treatments.

Together with Paula Clarke, we set out to compare three intervention programmes to promote reading comprehension skills which were specifically designed for poor comprehenders (Clarke *et al.* 2010). Building on the evidence that interventions which target metacognitive strategies involved in text comprehension can be effective, the first intervention involved text level training within the written language domain (text comprehension, TC). The second intervention took as its starting point the finding that poor comprehenders exhibit a range of oral language difficulties beyond phonology and hence included training activities to promote vocabulary, figurative language and listening comprehension skills (oral language, OL). Finally, an integrated programme was devised that incorporated activities to improve oral language and to enhance text level processing. This intervention (combined, COM) contained all of the activities in the TC and OL programmes but was specially designed to foster an integrative approach. The children with reading comprehension impairment who took part in this trial were selected by screening about 1000 children in 20 Year 4 classes. In each class, the eight children with the weakest comprehension skills in the presence of adequate decoding were selected. They were then allocated randomly to one of four groups, either to receive one of the interventions or to participate as a waiting list control who would receive the intervention at the end of the trial.

When designing these programmes, the intention was to make the text comprehension (TC) and oral language (OL) programmes as parallel as possible with the former delivered through written language and the latter through spoken language. In practice, this is not feasible because some activities lend themselves very directly to one modality or the other. Accordingly, the programmes were similar in that they each contained four components, but not all of the components were parallel. Both programmes built upon strategies from the reciprocal teaching approach whereby children worked together with their tutor to reflect on their comprehension of a text and in the discourse aimed to clarify and summarize their understanding, to generate questions and to make predictions. In the TC arm this strategy was used with written texts and in the OL programme in relation to listening. The two programmes were also parallel in their inclusion of narrative which was taught through writing stories in the TC intervention and through telling stories in the OL intervention. The other two components of each intervention differed. In the TC programme there was work on metacognitive strategies and inferencing from text, whereas in the oral language intervention there was explicit teaching of vocabulary using a multiple context approach (Beck *et al.* 2002) as well as activities to promote knowledge of figurative language. The combined programme (COM) included all eight components with children given opportunities to learn new vocabulary and idioms and to use inferences in both written and spoken language (for further details, see http://www.readingformeaning.co.uk).

The three different interventions to promote reading comprehension were delivered by trained teaching assistants in three sessions each week, two involving children working in dyads and one being an individual session. The teaching assistants were supported in fortnightly tutorials and they also each received a visit during which their teaching was observed and feedback was provided. The children receiving the interventions and the children in the waiting control group were assessed at pre-intervention (Time 1), 10 weeks later at mid-test (T2), and 10 weeks later at the end of the intervention post-test (T3). The children were also followed for 11 months before they were reassessed to investigate maintenance of gains. After this follow-up testing the children in the waiting list control group received a version of the COM programme delivered by the same teaching assistants for 20 weeks.

**Figure 1:**
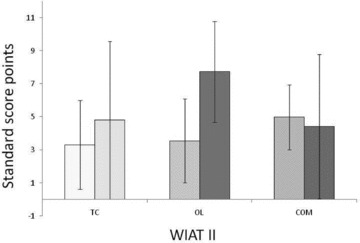
Gains in reading comprehension made by children receiving the text comprehension (TC), oral language (OL) and combined (COM) approaches relative to ‘waiting’ controls (on the Wechsler Individual Achievement Test (WIAT-II) at immediate post-intervention (light bars) and 11 months later (dark bars)).

Figure 1 shows the gains made in standard score points by the children in the three intervention conditions compared with progress made by the waiting list controls, controlling in each case for baseline performance. As shown, all three interventions brought about significant gains in text comprehension after 20 weeks of training. The average gain in standard scores was 3 points, which is small but statistically significant. Figure 1 also shows the scores of the children when assessed 11 months later at follow-up. All the gains remained significant, but strikingly the performance of the children in the oral language intervention was now significantly ahead of that of the children in the TC and COM arms, indicating that they had made a gain of some 7 standard score points over that of controls.

In summary, for children who reach middle-school age and have specific difficulties with reading comprehension, a number of different approaches appear to be effective. Children showed benefits when following a systematic intervention programme delivered through the written modality, incorporating activities to improve reading comprehension strategies, inferencing skills, and the understanding of story structure and narrative (TC). Similar benefits on reading comprehension can be observed following an oral language intervention that focuses on promoting listening comprehension, vocabulary, figurative language and oral narrative skills (OL). Moreover, there are equal benefits of combining the two approaches. However, in terms of sustained benefits, the oral language approach shows the most promising results in that the children who received this programme continued to forge ahead after the intervention ceased.

It is intriguing to consider what caused the sustained effects of the OL programme on reading comprehension after the end of the treatment. A further statistical analysis suggested that gains in vocabulary skill at Time 3 partially mediated developments in reading comprehension at Time 4 for children who received the OL programme. A simple explanation of this finding is that growth in vocabulary increases an individual's ability to understand not only single words, but also sentences and arguably increases resources that are available for making inferences across the text. An alternative view might be that children who receive oral language training become somehow more engaged with learning and this accounts for the further gains which they made.

The findings of this intervention trial have a number of theoretical implications. First text-level intervention is an effective strategy for promoting reading comprehension and furthermore, the effect is specific to reading and not mathematics. This finding underlines the efficacy of text comprehension approaches and suggests that the inefficient use of reading strategies may be one cause of reading comprehension failure. However, perhaps somewhat counter-intuitively, oral language interventions also impact reading comprehension and their impact appears to be at least partially mediated by vocabulary growth. This finding suggests that oral language difficulties are a causal risk factor for reading comprehension impairment and perhaps more specifically vocabulary deficits may be a critical causal factor.

### Early intervention to promote the foundations of reading comprehension

There are now a number of longitudinal studies that have followed the progress of children learning to read and have used retrospective analysis to ascertain the characteristics of children who go on to have reading comprehension difficulties (Catts *et al.* 2008, Nation *et al.* 2010). These studies show that problems of reading comprehension, and in particular the poor comprehender profile, are predated by impairment in oral language skills including vocabulary and grammar. Together with the results of the intervention described above, these findings suggest that oral language difficulties are a causal risk factor in reading comprehension impairment. This then is prima facie evidence to support the early implementation of programmes that foster oral language development in children with such difficulties. More generally, such difficulties will impact on a child's ability to listen in the classroom and to benefit from the education which they receive, much of which will be delivered initially through the spoken modality and later through written and textual materials.

We have already described an early intervention which we evaluated for children who enter school with poorly developed speech and language. It will be recalled that these children were randomly allocated to receive either the P+R programme or an alternative treatment. The alternative treatment was an OL programme and it is to this that we now turn. The OL programme comprised three main components: speaking and listening, vocabulary training and narrative work. It was delivered on a daily basis for 20 weeks alternating between small group and individual sessions. Further details of the content of the interventions are shown in Table 2. We examined the performance of these children on tests of oral language at the end of the intervention and 5 months later, comparing their performance with that of children who received the phonology P+R programme.

**Table 2:** Outline of oral language (OL) sessions (Bowyer-Crane *et al.* 2008)

Group session (30 min)	Individual session (20 min)
Introduction (5 min)	Introduction (2 min)
New vocabulary multi-sensory learning (5 min)	Vocabulary revision (5 min)
Vocabulary reinforcement (7 min)	Narrative task (5 min)
Speaking/listening/inferencing (10 min)	Listening, speaking and inferencing (5 min)
Plenary/best listener (3 min)	Plenary (3 min)

The OL intervention brought about pleasing gains in children speaking skills. There was evidence that the children had learned and retained the specific vocabulary that they had been taught during intervention and they now obtained significantly better scores on tests of expressive grammar than the P+R group. Children's narrative skills had also improved, though gains were only marginally significant. It was notable, however, that the impact of the intervention on a standardized test of expressive vocabulary and on a test of listening comprehension was not significant and the programme did not show any transfer to reading comprehension at this early stage in development. These findings have a number of implications. First, it is clearly possible to bring about gains in vocabulary and expressive language skills during the early school years. However, these gains do not directly transfer to improvements in reading skill (we can infer they might do if they were to incorporate specific work on letter, sounds and phonological awareness). It follows that poor language is not a direct cause of poor decoding ability though poor language is known to be a risk factor for more general reading difficulties (Snowling *et al.* 2000). The findings leave open the possibility that oral language training delivered prior to reading instruction within which both emergent language and literacy skills are brought together could be a more effective approach (for one example, see http://www.york.ac.uk/psychology/research/groups/crl/research/nuffield-language/).

## Implications for evidence-based practice

The present review has focused on randomized trials evaluating interventions that promote language and literacy for delivery in mainstream school settings. Underlying this work is the theoretical assumption that oral language is the foundation for written language skills; this tenet, embodied in the ‘Simple View of Reading’ (Gough and Tunmer 1986), formed the backdrop to the Rose (2006) review into the teaching of reading, which, in turn, led to the implementation of the phonic curriculum in English schools. Hence, according to Rose it is important to ensure that ‘best practice for beginner readers provides them with a rich curriculum that fosters all four interdependent strands of language: speaking, listening, reading and writing’ (p. 16). An obvious question that follows on from this assertion is really the nub of the present review: What therefore should be done for the child who comes to the task of reading with poorly developed oral language skills? More contentious perhaps: What are the roles of the different (health and education) professionals involved with these children?

From the perspective of research our view is straightforward. If a child is poorly prepared to learn to read and/or is experiencing reading difficulties, an integrated approach is vital. It may not matter who delivers an intervention; what matters more is that an evidenced-based intervention is chosen that fits the child's additional needs and that the person delivering it is properly trained and supported. In the independent review of ways to support children with dyslexia and other literacy difficulties Rose (2009) argued for a ‘3 waves’ approach. Some of the points made in this review are critical to the current discussion. Thus, if children are being taught using a systematic phonics approach, delivered within a language-rich curriculum (Wave 1), then teachers will be well positioned to identify early a child who is progressing more slowly than his or her peers. For such a child, the first step is to differentiate the curriculum within Wave 1. For children who need to ‘catch up’, a Wave 2 programme can be implemented, while a more individualized approach may be appropriate for those more severely affected.

Within this framework there are promising findings: there is good evidence that teachers can rate the progression of their pupils in phonics accurately, and that the children they judge to be progressing poorly show many of the characteristics of children with dyslexia (Snowling *et al.* 2011). As discussed above, our group has provided evidence of some effective catch-up programmes (for other examples, see Brooks 2007, and Duff and Clarke 2011).

However, why wait for failure? Indeed, Allen (2011) places early intervention before children fail at the heart of the political agenda, reinforcing a view that we share. Put simply, it is important to foster the development of oral language skills as a foundation for literacy development. Moreover, although Allen advocates ‘greater use of evidence based programs … and early intervention strategies that are proven to work’ (p. 67), not a single oral language intervention programme is listed within the recommendations of the report. Our discussion highlights an approach that can be effective for some children, and one which might be delivered by health and education services working together in the interests of the child.

## Conclusions

Language and phonological skills are the foundations of literacy development. Intervention programmes targeted to improve phonological skills and letter knowledge in at-risk children can be effective in promoting decoding skills during the early years and also in poor readers at later stages of development. At present there is good evidence that early intervention for oral language development can have positive effects, but as yet the evidence that such programmes affect longer-term reading outcomes is lacking. Notwithstanding this, children who have poor reading comprehension in the middle-school years can benefit from targeted intervention programmes that promote either text comprehension or oral language skills.

This review has distinguished reading disorders that affect decoding from reading disorders that affect comprehension. Arguably, however, this is a false dichotomy and in any school population the children who have pure decoding or pure comprehension difficulties will be small relative to those who have more general reading problems. In short, neither dyslexia nor reading comprehension impairment is a diagnostic entity with clear-cut boundaries (Rose 2009, Snowling 2009). It follows that targeted interventions need to focus on the dimensions that underpin disorders and in this endeavour both education and speech and language therapy have key roles to play.
